# Original Observation of Primary Bladder Histiocytic Sarcoma: First Case Report

**DOI:** 10.7759/cureus.12771

**Published:** 2021-01-18

**Authors:** Marta Nicola, Monica Onorati, Mauro Lancia, Virginia Varca, Franca Di Nuovo

**Affiliations:** 1 Pathology, ASST Rhodense, Garbagnate Milanese, ITA; 2 Urology, ASST Rhodense, Garbagnate Milanese, ITA; 3 Pathology, G. Salvini Hospital, Garbagnate Milanese, ITA

**Keywords:** histiocytic sarcoma, urinary bladder, differential diagnoses, histological features

## Abstract

Histiocytic sarcoma (HS) is a rare malignant lymphohematopoietic neoplasm; it has been cited in the recent World Health Organization (WHO) classification as a malignant proliferation of cells exhibiting morphological and immunophenotypic features of mature histiocytes. To our knowledge, the present case is the first to be described in the bladder of a patient without a history of lymphoma. Only one case has been reported so far regarding a secondary bladder presentation in the setting of a previous diffuse large B-cell lymphoma. We discuss the case of a 68-year-old male who presented with hematuria and dysuria. CT scan revealed a 4-cm intravesical mass that histological examination defined as HS. Our objective was to describe the clinical, histological, immunophenotypical, molecular characteristics and discuss the differential diagnoses of this first case of primary bladder HS. Our research was based on a review of selected articles obtained via the PubMed database. This extremely rare experience provided us with the opportunity to depict an interesting case, highlight its uniqueness, and build up new pathological evidence.

## Introduction

Histiocytic sarcoma (HS) is a rare malignant lymphohematopoietic neoplasm, which has been classified by the World Health Organization (WHO) as a malignant proliferation of cells exhibiting morphological and immunophenotypic features of mature tissue histiocytes [1]. The term histiocytic sarcoma was introduced in 1970, and since then, it has been widely used in many cases of hematolymphoid neoplasms with an epithelioid or histiocytoid morphology, including several cases misdiagnosed as large B‐cell lymphoma and anaplastic large cell lymphoma [2]. The use of immunohistochemistry (IHC) has improved the knowledge of this entity and helped to exclude other mimics, confirming its histiocytic origin and its rarity. Consequently, current literature data estimate that <1% of tumors presenting in lymph nodes or soft tissue can be defined as HS [3].

Even though HS can occur in people of all ages and has a slight male predominance, it is usually seen in adults. Cases reported in the literature so far have described presentation in lymph nodes, skin, and other extranodal sites, including the gastrointestinal tract, superficial and deep soft tissue, lungs, and nasal cavity [2]. Only one case of secondary bladder presentation in the setting of a previous large diffuse B-cell lymphoma has been described to date [4].

Histologically, the neoplasm consists of epithelioid and pleomorphic cells with abundant eosinophilic cytoplasm, often with some fine vacuoles. These atypical cells show large, round to oval nuclei, sometimes placed at the periphery of the cytoplasm with vesicular chromatin. The cellular background comprises a prominent inflammatory infiltrate consisting of small lymphocytes, plasma cells, benign histiocytes, neutrophils, and eosinophils. A definitive morphological diagnosis of HS is difficult, and it must be verified by IHC. The tumor cells express one or more tissue cell antigens, including CD163, CD68, CD45, and lysozyme, and they are usually negative for CD1a, langerin, CD21, CD35, CD13, and myeloperoxidase (MPO). Sometimes, the neoplasm could show positivity for CD15, pS-100, and CD4. HS mimics other epithelioid neoplasms such as lymphomas with large cell morphology, carcinoma, melanoma, and certain sarcomas. BRAF p.V600E alteration and additional mutations of the RAS/mitogen-activated protein kinase (MAPK) and phosphatidylinositol 3‑kinase (PI3K)/protein kinase B (AKT) pathways have been detected with variable frequency in recent years [5-6]. Because of its aggressive nature, the difficulty in its diagnosis, and the absence of a specific therapy, the prognosis for patients with HS is extremely poor [2,7]. HS may arise as a primary neoplasm or can occur in the setting of a concomitant or metachronous hematologic malignancy (especially low-grade B-cell lymphomas) or mediastinal germ cell tumor [3,7-9].

Its clinical presentation as a primary urinary bladder neoplasm is very rare: actually, to our knowledge, this case is the first of its kind to be described. Only one case has been reported so far regarding a secondary bladder presentation in the setting of a previous large diffuse B-cell lymphoma [4]. Therefore, the aim of our report is to present in detail the clinical, histological, and immunophenotypical characteristics of a case of primary bladder HS and to discuss the importance of histological differential diagnosis.

## Case presentation

**Figure 1:**
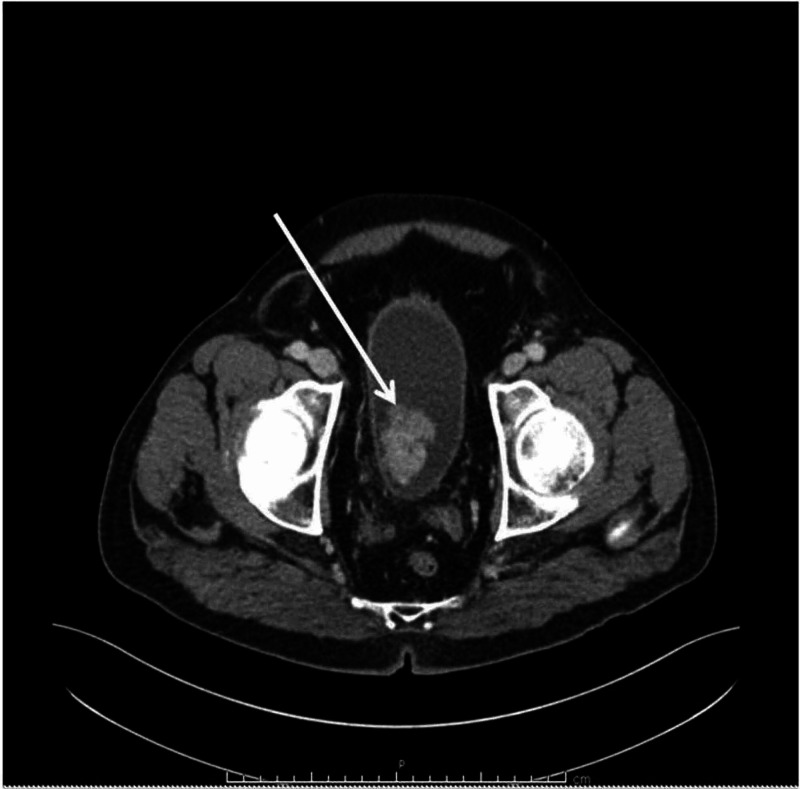
CT scan of the abdomen CT scan showing 4-cm heterogeneous intravesical mass, located at the posterior and lateral right wall of the urinary bladder (arrow) CT: computed tomography

**Figure 2:**
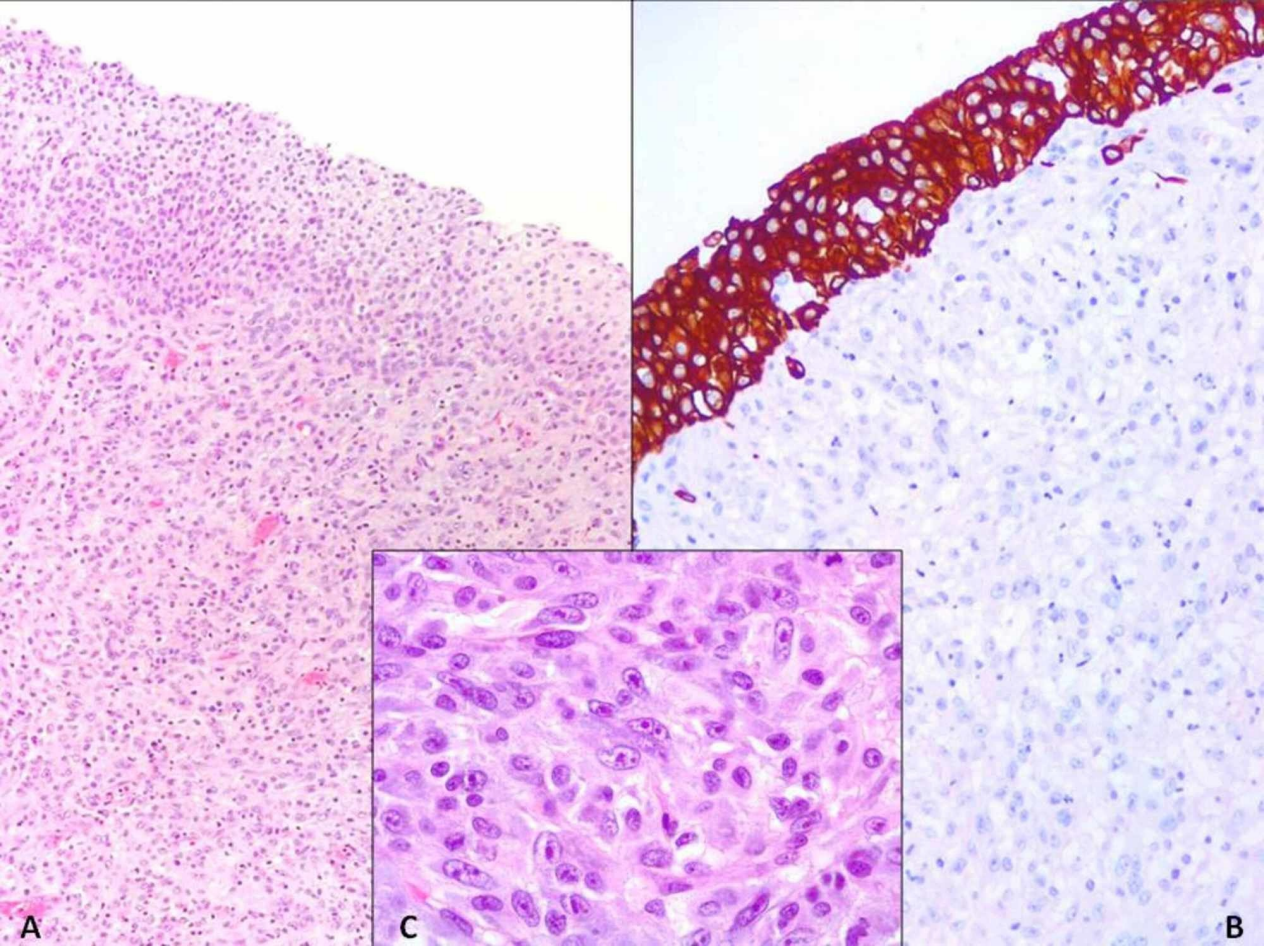
Histopathological characterization of TUR specimen A: bladder mucosa covered by normal urothelium; in the subepithelial connective tissue, there is a diffuse proliferation of atypical spindle cell (H&E, 10x); B: cytokeratin 7 highlighting normal urothelium (SABC, 20x); C: slightly spindled cells with amphophilic to finely vacuolated cytoplasm and with oval nuclei, with vesicular chromatin and fine nucleoli (H&E, 63x) TUR: transurethral resection; H&E: hematoxylin and eosin stain; SABC: Strept Avidin Biotin-peroxidase Complex Method

A 68-year-old male with a past medical history of hypertension presented to our hospital due to recurrent episodes of hematuria, dysuria, frequency, back pain, complaints of fever, fatigue, decreased appetite, and weight loss. Laboratory findings revealed anemia. Microbiology results were reported negative. Serial urine cytological examination was characterized by rare urothelial elements with slight cytological alterations. CT scan of the abdomen showed a 4-cm heterogeneous intravesical mass, located at the posterior and lateral right wall of the urinary bladder (Figure 1), which was confirmed by cystoscopy. The patient underwent transurethral resection (TUR) of the tumor that revealed a highly undifferentiated neoplasm composed of monomorphous, slightly spindled cells with amphophilic to finely vacuolated cytoplasm (Figure 2). These atypical cells show large, round to oval nuclei, sometimes placed at the periphery with vesicular chromatin and fine nucleoli (Figure 2). Occasionally, haemophagocytosis was observed; no multinucleated giant cells were detected. Several mitotic figures and wide areas of necrosis could be observed. A wide panel of IHC was set up and it oriented towards a mesenchymal origin of tumor cells. Since the neoplasm was highly hemorrhagic and localized only to the bladder, a cystoprostatectomy was performed. On gross examination, the urinary bladder showed a greyish-reddish, ulcerate mass, measuring 4 cm at a maximum diameter with a friable cut surface, covered by abundant necrotic and hemorrhagic material and infiltrating the inner half of the muscle. The mass was located at the posterior and left bladder wall. The prostate and terminal ureters were not involved.

**Figure 3:**
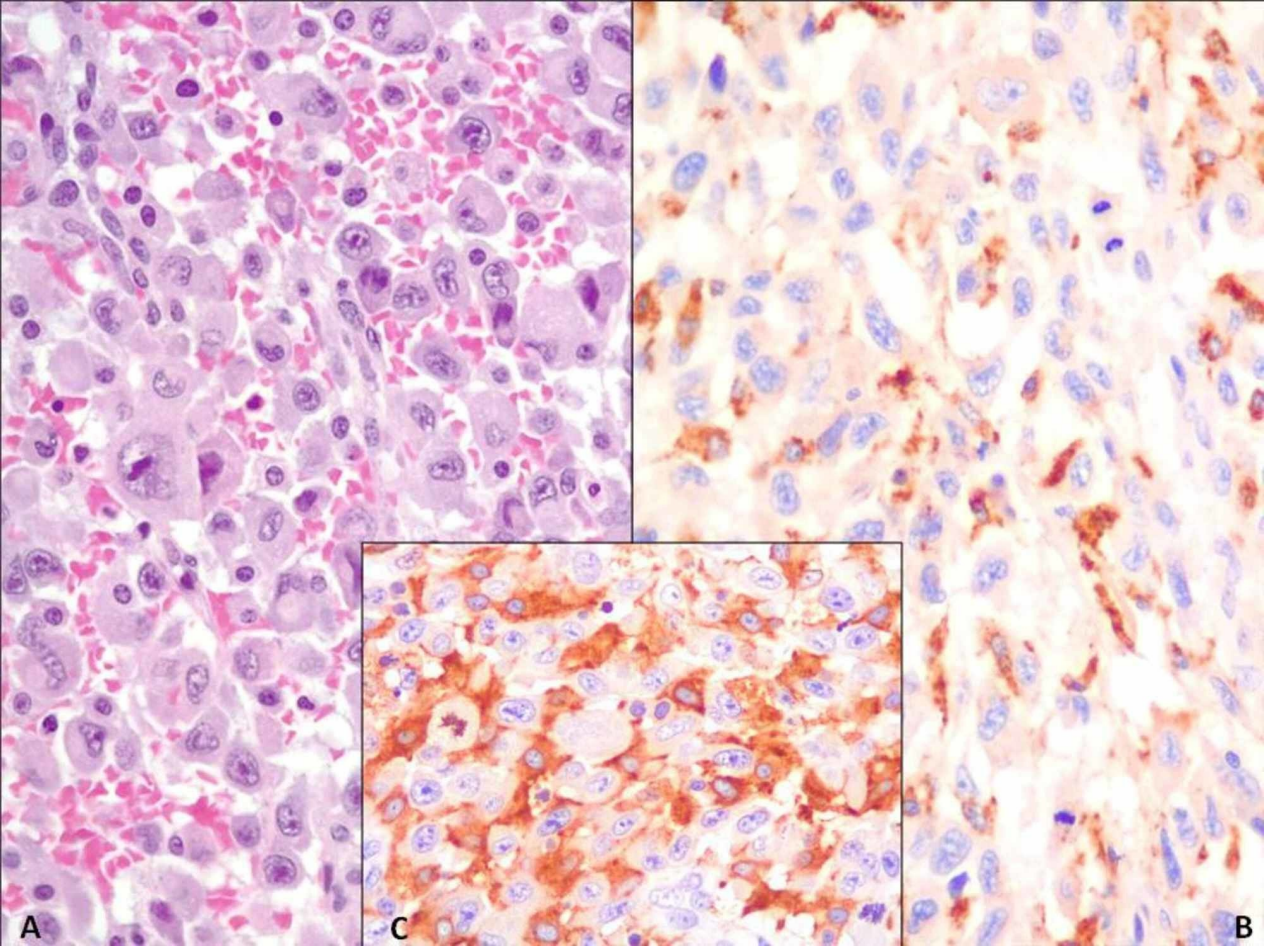
Histopathological characterization of cystoprostatectomy specimen A: non-cohesive, large, epithelioid, pleomorphic cells with finely vacuolated eosinophilic cytoplasm, with oval-shaped nuclei with dispersed chromatin and eosinophilic nucleoli (H&E, 40x); B: malignant epithelioid cells partially expressing CD68/KP1 (SABC, 40x); C: malignant epithelioid cells expressing histiocytic marker CD163 (SABC, 40x) H&E: hematoxylin and eosin stain; SABC: Strept Avidin Biotin-peroxidase Complex Method

Histologically, the neoplasm consisted of non-cohesive, large, epithelioid, pleomorphic cells with eosinophilic cytoplasm often with fine cytoplasmic vacuoles. Nuclei were mostly oval-shaped with dispersed chromatin and eosinophilic nucleoli; occasionally indented, slightly reniform nuclei could be detected (Figure 3). A high mitotic rate was observed. The cellular background was composed of a prominent inflammatory infiltrate consisting of small lymphocytes, plasma cells, benign histiocytes, neutrophils, and eosinophils. A wide panel of IHC reactions was performed: CD163, CD68/KP1, CD31, CD4, CD43, LCA, MPO, CD45, CD15, cytokeratin 7, GATA3, SMA, CD1a, pS-100, EMA, Ki67, CD21, CD23, CD20, CD3, ALK, bcl2 (Ventana) and CD68R/PGM1 (Dako). The tumor cells expressed the following antigens: CD163, CD68/KP1, CD4, and focally CD31, MPO, and CD68R/PGM1 (Figure 2). The proliferative index (Ki67) was about 70% of the neoplastic cells. The positivity of IHC histiocytic markers such as CD163, CD68/KP1, CD68R/PGM1, and CD4, in combination with cellular morphology, supported the histological diagnosis of HS. The other markers were fundamental for the differential diagnosis, in order to rule out other large cell neoplasms such as large cell lymphoma, melanoma, sarcoma, and carcinoma that can mimic HS. Moreover, we tested histological material for BRAF (exon 15), NRAS (exons 2, 3, 4), and KRAS (exons 2, 3, 4) mutations by molecular biology, and no genetic alterations were detected.

The patient’s clinical condition rapidly deteriorated after the surgical procedure with the quick onset of recurrent pelvic mass and with pulmonary and hepatic localization. Moreover, he developed sepsis and a leukemoid reaction and died within a few weeks. An autopsy was not performed.

## Discussion

The presentation of HS as primary urinary bladder neoplasm is very rare, and hence its diagnosis can be extremely complicated both on clinical and pathological grounds. In fact, to our knowledge, the present case is the first of its kind to be described. HS is an extremely rare hematological neoplasm with very few numbers of reported series, and it is grouped by the last WHO Classification of Tumours of Haematopoietic and Lymphoid Tissue in the chapter about histiocytic and dendritic cell neoplasms [1]. As previously stated, patients affected by this neoplasm display a wide age range, but most of the cases occur in adults with a slight male predilection [2,7].

This neoplasm may have nodal or extranodal involvement. The gastrointestinal tract, skin, liver, and soft tissue represent the most common sites of extranodal localization. Nodal localization and disseminated disease are less frequent [2,7]. Several studies have reported an association with other previous or simultaneous hematologic malignancies, such as low-grade B-cell lymphomas or B- or T-cell lymphoblastic leukemia, and rarely with mediastinal germ cell tumors. In these cases, HS and previous or concomitant hematologic malignancies share the identical clonal alteration, thereby supporting the hypothesis of transdifferentiation of the same malignant hematologic progenitor [8-11].

In this setting (i.e., history of diffuse large B-cell lymphoma), there has been only one other report of bladder involvement in HS to the best of our knowledge, and it involves an 80-year-old man [4]. However, our patient displayed an uneventful medical history, with the exception of hypertension, reported urinary symptoms, and a sole bladder mass was detected.

HS is difficult to diagnose because of both its rarity and its morphology that can mimic other more common neoplasms. The differential diagnoses include many different tumors, mostly with a large epithelioid or pleomorphic cell pattern. Lymphoproliferative disorders that can mimic HS include anaplastic and large cell non-Hodgkin's lymphoma, Hodgkin's lymphoma, follicular dendritic cell sarcoma, and Langerhans cell sarcoma. Among epithelial neoplasms, poorly differentiated or undifferentiated carcinomas must also be included. Moreover, HS can pose problems of differential diagnosis with melanoma and with some mesenchymal neoplasms like epithelioid angiosarcoma, epithelioid sarcoma, pleomorphic rhabdomyosarcoma, and unclassified pleomorphic sarcomas. In this setting, an immunohistochemical approach is mandatory to differentiate all these entities from HS [7]. Our case displayed different morphologies: in the TUR specimen, slightly spindled cells could be observed and only the presence of finely vacuolated cytoplasm could point towards a histiocytic origin in the absence of epithelioid, multinucleated giant cells, and of foamy cytoplasm. In the surgical specimen, an epithelioid morphology was easily appreciated with large, pleomorphic elements. However, the use of IHC markers is essential to prove the expression of specific histiocytic markers, such as CD163, CD68, and the lack of other lineage markers [12]. In our case, the neoplastic population expressed CD163, CD68/KP1, CD4, and focally CD68R/PGM1, MPO, and CD31. Likewise, epithelial markers, like cytokeratin 7 and GATA3, and melanocytic markers, such as p-S100, were negative. Given the condition's rarity and histologic overlap with different mimics, pathologists must bear in mind that histiocytic neoplasms do exist, although rare, and differential diagnosis can be extremely challenging.

The prognosis of HS is usually poor with the majority of patients dying of the progressive disease. Due to the infrequency of HS, the therapeutical experience is limited and no standard therapy has been properly established: in localized disease, surgical excision is used [2,12]. In our patient, persistent and severe hematuria, as well as the demonstration of a localized disease at presentation, prompted the use of cystoprostatectomy. However, recent reports of dramatic responses to adenosine-triphosphate-competitive inhibitors of BRAF kinase (i.e., vemurafenib and dabrafenib) alone or in combination with mitogen-activated protein kinase kinase (MEK) inhibitor trametinib, in the subset of BRAF-mutated HS, are paving the way to targeted/tailored therapy [10-11]. In our patient, targeted therapy was not attempted because no druggable genetic alterations (BRAF, NRAS, and KRAS) was found. The rapid deterioration in the patient’s clinical conditions did not allow for systemic therapy.

A part of the present article has been presented as an abstract at the Società Italiana di Anatomia Patologica (SIAPEC-IAP) Italian Congress of Pathology in 2019 in Torino, Italy.

## Conclusions

HS is a very rare malignant lymphohematopoietic neoplasm originating from mature tissue histiocytes, localizing both at nodal and extranodal sites without bone marrow involvement. Several cases have been reported in the setting of a previous or concomitant lymphoproliferative disease; however, only one report of primary bladder localization has been described after the occurrence of diffuse large B-cell lymphoma so far. Our report is the first of its kind of primary bladder involvement in HS without prior lymphoma. HS is an aggressive neoplasm with a dismal prognosis and, due to its infrequency, the therapeutical experience is limited and no standard therapy has been properly established. In conclusion, this extremely rare diagnostic experience gave us the opportunity to describe an unusual presentation of HS in order to build up new pathological and clinical evidence.
